# Intramedullary spinal cord abscess associated with right-to-left shunt via right superior vena cava draining into left atrium: A case report

**DOI:** 10.1097/MD.0000000000029740

**Published:** 2022-06-30

**Authors:** Satoshi Hirose, Naohiro Sudo, Masahiro Okada, Naotoshi Natori, Takayoshi Akimoto, Makoto Hara, Hideto Nakajima

**Affiliations:** a Division of Neurology, Department of Internal Medicine, Nihon University School of Medicine, Tokyo, Japan; b Department of Radiology, Nihon University School of Medicine, Tokyo, Japan.

**Keywords:** abscess, central nervous system infection, right-to-left shunt, spinal cord, superior vena cava

## Abstract

Intramedullary spinal cord abscess (ISCA) is a rare but treatable bacterial infection of the central nervous system, and the etiology in no less than 40% of the cases is cryptogenic. Although a few cases of ISCA in individuals with a right-to-left shunt (RL shunt) have been reported, only few arguments focused on the association between RL shunt and ISCA have been provoked. The right superior vena cava (RSVC) draining into the left atrium (LA) is an uncommon systemic venous anomaly that results in an RL shunt, and this anomaly causes several types of neurological complication such as stroke or brain abscess. We report the first case of ISCA associated with RSVC-LA RL shunt.

**Patient concerns:**

A 36-year-old man developed progressive paraparesis, dysuria, and spontaneous pain in the lumbar region and lower extremities. Spinal magnetic resonance imaging revealed an intramedullary lesion extended from Th12 to L2 with ring-shaped gadolinium enhancement. Cerebrospinal fluid (CSF) study exhibited a marked pleocytosis, and CSF culture grew *Streptococcus intermedius*. Cardiovascular computed tomography angiography identified RSVC-LA RL shunt, which caused transient acute cardiac syndrome due to air embolus.

**Diagnoses:**

The patient was diagnosed with ISCA associated with an RSVC-LA RL shunt.

**Interventions:**

The patient was treated with a combination of intravenous administration of meropenem and vancomycin in a daily dose of 6 and 2.5 g, respectively, followed by intravenous administration of ampicillin in a daily dose of 750 mg. The intravenous antibiotic therapy was continued for 37 days.

**Outcomes:**

A favorable neurological outcome was obtained by the intravenous antibiotic therapy, and recurrence of infection was prevented by continuous oral antibiotic therapy for 18 months.

**Lessons:**

With a literature review of ISCA associated with RL shunt, we insist that screening for RSVC-LA is beneficial to patients who are diagnosed with cryptogenic ISCA as its identification leads to appropriate preventive therapy.

## 1. Introduction

Intramedullary spinal cord abscess (ISCA) is a rare bacterial infection of the central nervous system (CNS) that can lead to sensory, motor, and/or autonomic dysfunction and is sometimes fatal.^[1]^ Although the etiology is cryptogenic in 40% of the cases,^[1]^ rare cases of ISCA in individuals with a right-to-left shunt (RL shunt) have also been reported.^[2–4]^ The right superior vena cava (RSVC) draining into the left atrium (LA) is an uncommon systemic venous anomaly that results in an RL shunt.^[5]^ This RSVC-LA RL shunt is typically identified in the neonatal period or childhood because of hypoxemia; however, hypoxemia, stroke, myocardial infarction, and brain abscess are seen in completely asymptomatic adult patients with normal growth and development.^[6–8]^ Here, we describe the first case of ISCA with an RSVC-LA RL shunt.

## 2. Case presentation

A 36-year-old Japanese man was admitted to our hospital with progressive paraparesis, dysuria, and spontaneous pain in the lumbar region and lower extremities that occurred 7 days before admission. He had a medical history of an endoscopic polypectomy performed for colonic polyps. His history was otherwise unremarkable. A neurological examination at admission revealed that he was alert and well oriented, and did not exhibit aphasia or cranial nerve involvement. However, extremely impaired perceptions to pinprick and vibration below the L2 level were noted, along with severe weakness of the lower extremities, predominantly in the distal regions. Tendon reflexes of the lower extremities were lost, and Babinski sign was absent in both sides. Nuchal rigidity was not present. He was unable to micturate. There was no fever or any other signs of systemic infection, and otorhinolaryngologic and dental evaluation did not reveal any signs of infection.

**Figure 1.**
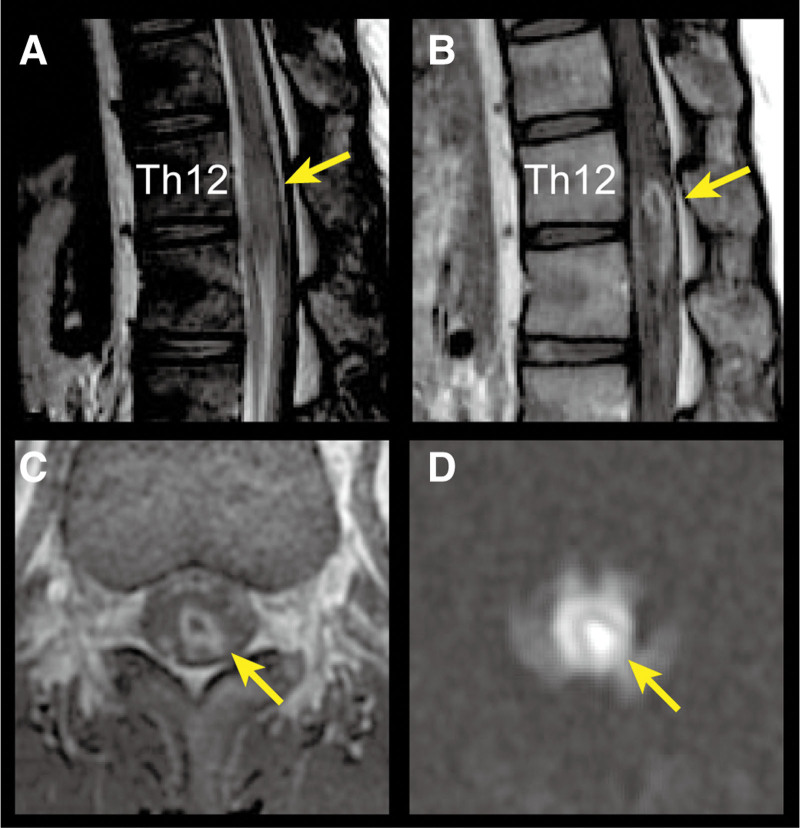
Spinal MRI. (A) Sagittal section of T2-weighed image of the thoracolumbar spine shows a high-intense area located at vertebral levels Th12–L2, associated with swelling of the spinal cord around the lesion, indicated by a yellow arrow. (B) Gadolinium-enhanced T1-weighted image shows enhancement around the lesion, indicated by a yellow arrow. (C) A ring-shaped gadolinium enhancement around the lesion in transverse section, indicated by a yellow arrow. (D) High-intensity lesion on diffusion-weighted image in transverse section, indicated by a yellow arrow. MRI = magnetic resonance imaging.

Spinal magnetic resonance imaging (MRI) (Fig. 1) revealed a high-intensity intramedullary lesion on T2-weighted imaging that extended from the Th12 to L2 vertebral levels (Fig. 1A), which appeared ring-shaped with gadolinium enhancement (Fig. 1B and C). Additionally, swelling of the spinal cord around the lesion was evident (Fig. 1A–C). The lesion exhibited high intensity on diffusion-weighted imaging (Fig. 1D) but low intensity on the apparent diffusion coefficient map. An enhanced whole-body computed tomography (CT) was unremarkable. A spinal tap yielded yellow and turbid cerebrospinal fluid (CSF) with a markedly high white blood cell count (6400/µL, polymorphonuclear cells 100%) and total protein level (357 mg/dL), along with low glucose (23 mg/dL; blood glucose 104 mg/dL). CSF was negative for both herpes simplex virus DNA and varicella zoster virus DNA upon high-sensitive polymerase chain reaction. Further, even though CSF culture grew *Streptococcus intermedius*, two sets of blood cultures remained sterile. Hematological analysis showed slightly elevated white blood cell count (10,900 cell/µL), normal C-reactive protein level, and no antibodies in the serum against aquaporin-4 or other systemic autoimmune diseases, including antinuclear, anti-double-stranded DNA, antiphospholipid, anti-Sjögren syndrome antigen A, anti-Sjögren syndrome antigen B antibodies, and anti-neutrophil cytoplasmic antibody. Urinalysis was normal.

**Figure 2.**
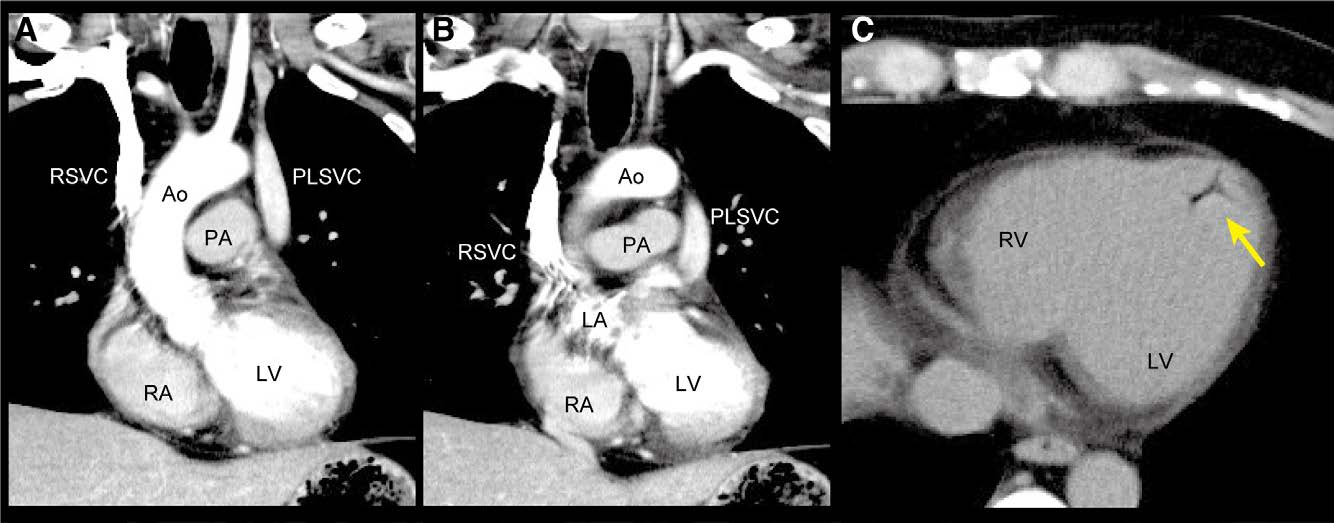
Cardiovascular CT angiography. (A, B) Coronal sections of contrast-enhanced images show the venous anomaly of the RSVC draining into the LA. (C) Transverse section of a noncontrast image shows air in the left ventricle, indicated by a yellow arrow. Ao = aorta, CT = computed tomography, LA = left atrium, LV = left ventricle, PA = pulmonary artery, PLSVC = persistent left superior vena cava, RA = right atrium, RSVC = right superior vena cava, RV = right ventricle.

Based on the above, he was diagnosed with ISCA and treated with a combination of intravenous administration of meropenem and vancomycin in a daily dose of 6 and 2.5 g, respectively, from the day of admission. Antibiotics were deescalated to ampicillin in a daily dose of 750 mg on day 9 based on antibiotic sensitivity of *S. intermedius* identified in the CSF. On day 14 after admission, he developed sudden onset chest tightness and decreased oxygen saturation with ST-segment elevations in leads II, III, and augmented vector foot on 12-lead electrocardiogram (ECG). A CT angiography under the pulmonary embolism protocol was performed with contrast medium injected through an intravenous catheter in the right forearm. Surprisingly, contrast was detected in the superior vena cava, the LA, the left ventricle (LV), and the aorta, but not in the pulmonary arterial system (Fig. 2A and B), which indicated the presence of an RL shunt.^[8]^ Additionally, air was detected in the LV (Fig. 2C), along with the following systemic venous anomalies (Fig. 2A and B), viz., a persistent left superior vena cava (PLSVC) that drained into the right atrium via the coronary sinus, the RSVC draining into the LA, and a defective brachiocephalic vein. The course of the inferior vena cava was normal. After a few hours, chest tightness disappeared, oxygen saturation increased, and a repeat ECG revealed no ST-segment elevations; hence, these symptoms were attributed to a transient acute cardiac syndrome caused by an air embolus during intravenous catheter placement in the right forearm that had migrated to the LA via drainage from the RSVC and subsequently reached the coronary arteries.

Follow-up CSF studies on days 4, 14, 25, 37, and 50 showed improvement in pleocytosis and decreased glucose, and MRI acquired on day 35 revealed resolution of the intramedullary lesion and no swelling of the spinal cord. No adverse event occurred during the intravenous antibiotic therapy, and the intravenous antibiotic therapy was discontinued on day 37. Continuous oral administration of ampicillin in a daily dose of 750 mg was then started to prevent recurrence of infection. The patient showed gradual improvement, was able to walk independently, and could return to work by 5 months after rehabilitation. At 18 months after disease onset, his muscle strength was normal, but urinary retention and rectal dysfunction persisted. During this follow-up period, no infectious diseases or exacerbation of neurological symptoms was seen. The patient provided written informed consent for publication of this case report.

## 3. Discussion

We describe the case of a 36-year-old man who developed progressive paraparesis, dysuria, and spontaneous pain in the lumbar region and lower extremities, and was diagnosed with ISCA associated with an RSVC-LA RL shunt. Antibiotic therapy successfully improved his neurological symptoms, and a favorable outcome was obtained, although urinary retention and rectal dysfunction remained. Recurrence of infection and neurological symptoms was prevented by continuous oral antibiotic therapy for 18 months.

ISCA is a rare bacterial infection of the CNS that leads to sensory, motor, and/or autonomic dysfunction, and may sometimes be fatal.^[1]^ A previous review of 54 cases of ISCA has reported that the cause of infection was cryptogenic in 39% of the patients, followed by congenital dermal sinus infection in 28%, sepsis in 26%, extension from contiguous lesions (e.g., diskitis, trauma, or surgery) in 6%, and infectious endocarditis in 1%.^[1]^ In our case, none of these common causes of ISCA was seen; rather, an anomalous RSVC-LA RL shunt was identified. ISCA in individuals with an RL shunt has been reported in three case reports in the English-language literature (Table 1)—one case with pulmonary arteriovenous fistula (PAVF)^[2]^ and two with patent foramen ovale (PFO).^[3,4]^ Although prevention therapy and neurological outcomes vary across these cases with ISCA and RL shunt (Table 1), a relatively favorable outcome was obtained using intravenous antibiotic and continuous oral antibiotic therapy in our patient.

**Table 1:** Cases of intramedullary spinal cord abscess associated with right-to-left shunt

Case no.	1	2	3	4
Author	David et al 1997^[2]^	Higuchi et al 2011^[3]^	Terterov et al 2011^[4]^	Our case
Age (yr), sex	27 M	51 M	59 M	36 M
Symptoms (mRS at peak)	Fever, tetraplegia, and sensory deficit (5)	Fever, tetraplegia, ischuria, constipation, dysphagia, and hiccup (5)	Tetraplegia and sensory deficit (5)	Paraplegia, urinary retention, and fecal incontinence (5)
Location	C5	Entire spinal cord and medulla oblongata	C3–C7	Th12–L2
Pathogens	*Haemophilus aphrophilus* and *Actinomyces meyeri*	*Streptococcus viridans*	*S viridans*	*Streptococcus intermedius*
RL shunt	PAVF	PFO	PFO	RSVC to LA
Treatment	Drainage of abscess and antibiotics	Antibiotics and corticosteroids	Antibiotics, corticosteroids, and surgical removal of abscess	Antibiotics
Prevention	Fistula embolization	None	Interventional cardiology on PFO, IVC filtering	Continuous oral antibiotics
Follow-up (mo)	N.A.	3	N.A.	16
Disability remained	“Satisfactory improvement”	Muscle weakness (able to walk with parallel bars)	Muscle weakness (MRC 3 in right side and MRC 1 in left side)	Urinary retention and fecal incontinence
Outcome (mRS)	1	3	4–5	1

IVC = inferior vena cava, LA = left atrium, MRC = Medical Research Council’s scale, mRS = modified Rankin Scale, N.A. = not available, PAVF = pulmonary arteriovenous fistula, PFO = patent foramen ovale, RL shunt = right-left shunt, RSVC = right superior vena cava.

A mechanism by which ISCA occurred in a case with PFO was speculated by Higuchi et al^[3]^ as follows; the presence of an RL shunt allows pathogens to bypass pulmonary defenses and enter the arterial circulation easily, which leads to high risk of ISCA as well as brain abscess. Brain abscess is a more common CNS infection than ISCA in the presence of an RL shunt, such as a PFO,^[9]^ cyanotic cardiac disease,^[10]^ or PAVF.^[11]^ Although less frequent, cases of brain abscess associated with RSVC-LA have also been reported.^[6,12,13]^ Bacteria are usually intercepted by phagocytosis in the pulmonary capillary vessels, but the RL shunt allows the bacterial mass to circumvent this, thereby permitting cerebral embolization of the bacterial mass and formation of a brain abscess.^[9,10,12]^ Interestingly, brain abscesses have been reported to occur without bloodstream infection in the presence of an RL shunt.^[6,11]^ Based on the aforementioned discussion, we suggest a mechanism by which ISCA could have occurred in our patient with the RSVC-LA RL shunt is as follows; a bacterial mass of *S. intermedius*, which is normal commensal oral flora in humans,^[14]^ initially invaded capillary vessels from the oral cavity, migrated to the aorta via the RSVC-LA RL shunt, and finally reached the capillary vessels in the intramedullary spinal cord where embolization of the bacterial mass led to formation of the abscess,^[9,10]^ as with the cases of brain abscess associated with RSVC-LA.^[6,12]^

Anomalous systemic venous drainage into the LA is an uncommon cause of RL shunt and, if present, is most commonly due to the PLSVC draining into the LA.^[15]^ Thus, the RSVC-LA is extremely rare in the absence of other cardiac abnormalities.^[8]^ Since its first identification in 1956,^[16]^ there have been approximately 60 reported cases of RSVC-LA, and most patients are diagnosed in the neonatal period or childhood because the shunt leads to hypoxemia and cyanosis.^[5,8]^ However, in adult patients, even after complete asymptomatic normal growth and development, this systemic venous anomaly can cause hypoxemia,^[8]^ myocardial infarction,^[17]^ stroke or transient ischemic attack,^[7,15,17]^ and brain abscess,^[6,12,13,15]^ with the need for surgical correction of this systemic venous anomaly varying among individual cases.^[5]^

To the best of our knowledge, only 6 detailed case reports of neurological complications secondary to an RSVC-LA RL shunt have been published (Table 2) in the English-language literature: three cases of brain abscess^[6,12,13]^ and three cases of cerebrovascular disorder.^[7,15,17]^ Our article is the first case description of ISCA in a patient with an RSVC-LA RL shunt. Three cases were female, and the median age was 46.5 (range, 34–65) years. Past history of myocardial infarction was reported in two cases,^[6,17]^ and our patient similarly developed a transient acute cardiac syndrome caused by an air embolus, which led to identification of his RSVC-LA RL shunt. Past history of brain abscess was reported in one case.^[15]^ Remarkably, hypoxemia due to RL shunt was seen in three cases despite their normal growth and development.^[7,12,13]^ Unsurprisingly, symptoms, treatments, and outcomes vary among the cases^[6,7,12,13,15,17]^ according to their neurological complications and affected regions. To prevent further complications due to RL shunt, surgical intervention was performed in three cases,^[7,12,13]^ and oral antithrombotic therapy was undergone in one case.^[17]^ While our patient continues to take oral antibiotics to prevent recurrence of the abscess or systemic infection, surgical intervention was deemed unnecessary due to the absence of hypoxemia or heart failure. We also instructed him that an intravenous catheter must not be placed in his right arm to avoid a risk of accidental complications caused by air embolus.

**Table 2:** Detailed neurological complications associated with the right superior vena cava draining into the left atrium

Case no.	1	2	3	4	5	6
Author	Schick et al 1985^[6]^	Leys et al 1986^[12]^	Sadek et al 2006^[7]^	Hong et al 2011^[13]^	Clark and MacDonald 2015^[15]^	Karavassilis et al 2021^[17]^
Age (yr), sex	49 M	44 M	36 F	34 F	65 M	49 F
Past history (age)	MI (46)	None	None	None	BA (22)	Sickle cell trait, MI (49), and anachronic PE (49)
Symptoms	Headache	Fever, headache, nausea, and homonymous hemianopia	Headache, vertigo, nausea, vomiting, ataxia, and nystagmus	Fever, headache, and nuchal rigidity	Speech disturbance and left arm monoparesis	Headache and homonymous hemianopia
Hypoxemia	no	85 mm Hg of PaO_2_	Mild hypoxemia	80% of SpO_2_	No	No
Neurological complication	BA	BA	stroke	BM and BA	TIA	Stroke
Location	Right frontal lobe	Parietal lobe	Left PICA territory	Right frontal lobe	Not applicable	Left PCA territory
Treatment	Drainage and lobectomy	Antibiotics	Anticoagulation	Antibiotics	Not available	Antiplatelet and anticoagulation
Prevention	Not applicable	Surgical ligation of RSVC just above LA	Surgical repair	Surgical connection of RSVC to RA	None (surgery was declined)	Antiplatelet and anticoagulation
Outcome	Dead	Complete recovery	Not available	Recovered quickly	Complete recovery	Minimal disability for hemianopia
Subsequent complications	None	None	None	None	None	Thrombophlebitis and DVT

BA = brain abscess, BM = bacterial meningitis, DVT = deep vein thrombosis, LA = left atrium, MI = myocardial infarction, PCA = posterior cerebral artery, PE = pulmonary emboli, PICA = posterior inferior cerebellar artery, RA = right atrium, RSVC = right superior vena cava, TIA = transient ischemic attack.

In summary, the RSVC-LA draining into the LA is a rare venous anomaly that can potentially lead to not only hypoxemia and cardiac events but also result in neurological complications due to the RL shunt. This case identifies RL shunt as an important etiological agent of ISCA, and hence, screening for RSVC-LA RL shunt may be beneficial to patients with cryptogenic ISCA as its identification can lead to appropriate preventive therapy.

## Author contributions

**Conceptualization:** Satoshi Hirose, Makoto Hara

**Data curation:** Satoshi Hirose, Naohiro Sudo, Naotoshi Natori,  Takayoshi Akimoto

**Funding acquisition:** Makoto Hara

**Investigation:** Satoshi Hirose, Naohiro Sudo, Masahiro Okada,  Naotoshi Natori, Takayoshi Akimoto, Makoto Hara, Hideto Nakajima

**Supervision:** Makoto Hara, Hideto Nakajima

**Visualization:** Satoshi Hirose

**Writing—original draft:** Satoshi Hirose

**Writing—review & editing:** Naohiro Sudo, Masahirose Okada,  Naotoshi Natori, Takayoshi Akimoto, Makoto Hara, Hideto Nakajima

## References

[1] Iwasaki M Yano S Aoyama T. Acute onset intramedullary spinal cord abscess with spinal artery occlusion: a case report and review. Eur Spine J 2011;20 Suppl 2:S294–301. 21308472 10.1007/s00586-011-1703-z PMC3111523

[2] David C Brasme L Peruzzi P. Intramedullary abscess of the spinal cord in a patient with a right-to-left shunt: case report. Clin Infect Dis Off Publ Infect Diseases Soc Am 1997;24:89–90. 10.1093/clinids/24.1.89 8994758

[3] Higuchi K Ishihara H Okuda S. A 51-year-old man with intramedullary spinal cord abscess having a patent foramen ovale. BMJ Case Rep 2011;2011:bcr1120103512. 10.1136/bcr.11.2010.3512 PMC3091272 22696715

[4] Terterov S Taghva A Khalessi AA. Intramedullary abscess of the spinal cord in the setting of patent foramen ovale. World Neurosurg 2011;76:361.e11361.e311–361.e14. 10.1016/j.wneu.2010.01.008 21986439

[5] Chowdhury UK Anderson RH George N. A review of the surgical management of anomalous connection of the right superior caval vein to the morphologically left atrium and biatrial drainage of right superior caval vein. World J Pediatr Congenit Heart Surg 2020;11:466–84. 32645787 10.1177/2150135120912677

[6] Schick EC Jr. Lekakis J Rothendler JA. Persistent left superior vena cava and right superior vena cava drainage into the left atrium without arterial hypoxemia. J Am Coll Cardiol 1985;5:374–8. 3968321 10.1016/s0735-1097(85)80063-2

[7] Sadek H Gilkeson RC Hoit BD. Images in cardiovascular medicine. Case of anomalous right superior vena cava. Circulation 2006;114:e532–3. 17030693 10.1161/CIRCULATIONAHA.106.626101

[8] Baggett C Skeen SJ Gantt DS. Isolated right superior vena cava drainage into the left atrium diagnosed noninvasively in the peripartum period. Tex Heart Inst J 2009;36:611–4. 20069093 PMC2801949

[9] Sadahiro H Nomura S Inamura A. Brain abscess associated with patent foramen ovale. Acta Neurochir 2014;156:1971–6. 24975278 10.1007/s00701-014-2170-1

[10] Lumbiganon P Chaikitpinyo A. Antibiotics for brain abscesses in people with cyanotic congenital heart disease. Cochrane Database Syst Rev 2013;2013:Cd004469. 10.1002/14651858.CD004469.pub3 PMC7197203 23543532

[11] Gao LY Xu GR Dai TJ. Precision diagnosis and therapy of a case of brain abscesses associated with asymptomatic pulmonary arteriovenous fistulas. BMC Infect Dis 2020;20:370. 32448130 10.1186/s12879-020-05092-6 PMC7247166

[12] Leys D Manouvrier J Dupard T. Right superior vena cava draining into the left atrium with left superior vena cava draining into the right atrium. Brit Med J (Clin Res Ed) 1986;293:855. 10.1136/bmj.293.6551.855 PMC1341636 3094684

[13] Hong SN Nayar A Srichai MB. A case of an anomalous superior vena cava with anomalous pulmonary veins-when two wrongs do not make a right. Echocardiography 2011;28:E39–41. 20678126 10.1111/j.1540-8175.2010.01255.x

[14] Issa E Salloum T Tokajian S. From normal flora to brain abscesses: a review of *Streptococcus intermedius*. Front Microbiol 2020;11:826. 32457718 10.3389/fmicb.2020.00826 PMC7221147

[15] Clark C MacDonald L. Right-sided superior vena cava draining into the left atrium in a patient with persistent left-sided superior vena cava emptying into the right atrium diagnosed by echocardiography. Proc (Bayl Univ Med Cent) 2015;28:365–6. 26130891 10.1080/08998280.2015.11929276 PMC4462224

[16] Wood PH. Diseases of the Heart and Circulation. 2nd ed. Philadelphia, PA: Lippincott 1956:457.

[17] Karavassilis ME Haji-Coll M Keenan NG. Multiple thromboembolic events associated with bilateral superior vena cava and anomalous drainage into the left atrium. BMJ Case Rep 2021;14:e237401. 10.1136/bcr-2020-237401 PMC7852915 33526519

